# Cilengitide: The First Anti-Angiogenic Small Molecule Drug Candidate. Design, Synthesis and Clinical Evaluation

**DOI:** 10.2174/187152010794728639

**Published:** 2010-12

**Authors:** Carlos Mas-Moruno, Florian Rechenmacher, Horst Kessler

**Affiliations:** Institute for Advanced Study, Department Chemie, Technische Universität München, Lichtenbergstrasse 4, 85747 Garching, Germany

**Keywords:** RGD peptides, integrin antagonists, glioblastoma, N-methylation, αvβ3, conformational restriction, cyclization.

## Abstract

Cilengitide, a cyclic RGD pentapeptide, is currently in clinical phase III for treatment of glioblastomas and in phase II for several other tumors. This drug is the first anti-angiogenic small molecule targeting the integrins αvβ3, αvβ5 and α5β1. It was developed by us in the early 90s by a novel procedure, the spatial screening. This strategy resulted in *c*(RGDfV), the first superactive αvβ3 inhibitor (100 to 1000 times increased activity over the linear reference peptides), which in addition exhibited high selectivity against the platelet receptor αIIbβ3. This cyclic peptide was later modified by *N*-methylation of one peptide bond to yield an even greater antagonistic activity in *c*(RGDf(*N*Me)V). This peptide was then dubbed Cilengitide and is currently developed as drug by the company Merck-Serono (Germany).

This article describes the chemical development of Cilengitide, the biochemical background of its activity and a short review about the present clinical trials. The positive anti-angiogenic effects in cancer treatment can be further increased by combination with “classical” anti-cancer therapies. Several clinical trials in this direction are under investigation.

## INTRODUCTION

Integrins are heterodimeric receptors that are important for cell-cell and cell-extracellular matrix (ECM) interactions and are composed of one α and one β-subunit [1, 2]. These cell adhesion molecules act as transmembrane linkers between their extracellular ligands and the cytoskeleton, and modulate various signaling pathways essential in the biological functions of most cells. Integrins play a crucial role in processes such as cell migration, differentiation, and survival during embryogenesis, angiogenesis, wound healing, immune and non-immune defense mechanisms, hemostasis and oncogenic transformation [1]. The fact that many integrins are also linked with pathological conditions has converted them into very promising therapeutic targets [3]. In particular, integrins αvβ3, αvβ5 and α5β1 are involved in angiogenesis and metastasis of solid tumors, being excellent candidates for cancer therapy [4-7]. 

There are a number of different integrin subtypes which recognize and bind to the tripeptide sequence RGD (arginine, glycine, aspartic acid), which represents the most prominent recognition motif involved in cell adhesion. For example, the pro-angiogenic αvβ3 integrin binds various RGD-containing proteins, including fibronectin (Fn), fibrinogen (Fg), vitronectin (Vn) and osteopontin [8]. It is therefore not surprising that this integrin has been targeted for cancer therapy and that RGD-containing peptides and peptidomimetics have been designed and synthesized aiming to selectively inhibit this receptor [9, 10]. 

One classical strategy used in drug design is based on the knowledge about the structure of the receptor-binding pocket, preferably in complex with the natural ligand. However, this strategy, the so-called “rational structure-based design”, could not be applied in the field of integrin ligands since the first structures of integrin’s extracellular head groups were not described until 2001 for αvβ3 [11] (one year later, in 2002 the structure of this integrin in complex with Cilengitide was also reported [12]) and 2004 for αIIbβ3 [13]. Therefore, initial efforts in this field focused on a “ligand-oriented design”, which concentrated on optimizing RGD peptides by means of different chemical approaches in order to establish structure-activity relationships and identify suitable ligands.

We focused our interest in finding ligands for αvβ3 and based our approach on three chemical strategies pioneered in our group: 1) Reduction of the conformational space by cyclization; 2) Spatial screening of cyclic peptides; and 3) *N*-Methyl scan.

The combination of these strategies lead to the discovery of the cyclic peptide *c*(RGDf(*N*Me)V) in 1995. This peptide showed subnanomolar antagonistic activity for the αvβ3 receptor, nanomolar affinities for the closely related integrins αvβ5 and α5β1, and high selectivity towards the platelet receptor αIIbβ3. The peptide was patented together with Merck in 1997 (patent application submitted in 15.9.1995, opened in 20.3.1997) [14] and first presented with Merck’s agreement at the European Peptide Symposium in Edinburgh (September 1996) [15]. The synthesis and activity of this molecule was finally published in 1999 [16]. This peptide is now developed by Merck-Serono, (Darmstadt, Germany) under the name "Cilengitide" and has recently entered Phase III clinical trials for treating glioblastoma [17].

The aim of this review is to describe the chemical development of Cilengitide in our laboratory, the biochemical background for its biological activity and to give a comprehensive summary of the clinical trials performed so far.

## 1 DISCOVERY OF CILENGITIDE: DESIGN AND SYNTHESIS

### 1.1 The RGD-Binding Motif

Pioneering studies by Ruoslahti and Pierschbacher in the early 1980s revealed the RGD motif as the cell attachment site within the Fn module and its crucial role in the interaction of Fn with its cell surface receptor [18-21]. These studies initially described the tetrapeptide sequence Arg-Gly-Asp-Ser (RGDS) as the minimal binding site of Fn [19], but shortly after it was demonstrated that the serine residue can be replaced by other amino acids without significant loss of biological function, whereas arginine, glycine and aspartic acid are totally essential for the activity [20].

Subsequently the RGD motif was found in other ECM proteins capable of binding to the integrin receptors (the term *integrin* was introduced by Hynes and coworkers in 1986 [22]) such as Vn, osteopontin, collagens, von Willebrand factor, Fg, thrombospondin and laminin [23-26]. Together with these findings, it was observed that although many integrins recognize the RGD motif, they are also able to discriminate among distinct natural ligands (ECM proteins) containing this same recognition motif [27]. Even though the presence of distinct amino acids flanking the RGD motif certainly contributes to receptor selectivity, this it is not enough to explain this behavior. To answer this question, it was postulated that integrin receptors are able to recognize distinct conformations of RGD sequences, which are maintained by the protein (secondary and tertiary) structure.

The concept of conformation-dependent recognition was supported by early studies showing that short peptides with the same sequence, displayed different conformations when inserted into unrelated proteins, and were in turn recognized by unique antibodies [28]. In this regard, the integration of a recognition motif into a cyclic peptide is a feasible way to restrict the conformational space of the amino acid sequence, and was demonstrated to show an impact on binding affinity and receptor specificity [29]. This concept, discussed by us already more than a quarter of a century ago, will be detailed in the next section.

### 1.2 Restriction of Conformation by Cyclization and Spatial Screening

Linear peptides possess an enormous number of conformations in solution. This flexibility does not necessarily mean absence of biological activity for these molecules, but it is often related to poor selectivity. A useful way to reduce the conformational space of linear peptides is cyclization [29, 30]. The restriction in a molecule’s conformational freedom may have positive effects in terms of binding affinity and selectivity to a receptor, provided that the biologically active conformation is allowed in the restrained conformational space (matched case). On the other hand, when the peptide is not able to adopt the bioactive conformation (mismatched case), the activity is considerably reduced or totally lost. The gain in biological activity for the matched situation is explained by the decrease in conformational entropy that is lost upon binding to the receptor, and by the pre-induced strain toward adoption of the bound conformation.

In this sense, a “promiscuous” behavior is expected for linear RGD-containing peptides in binding different integrin receptors, whereas constrained analogues may exhibit improved activity and selectivity profiles. This concept was proved by a disulfide cyclized synthetic RGD-peptide, which showed an improved inhibition of Vn-mediated adhesion and no inhibitory activity for Fn adhesion, compared to the unselective stem linear peptide [31]. It was also reported that reduction of disulfide bridges in several snake venom RGD-containing peptides, the disintegrins, significantly decreased their platelet aggregation inhibitory activity [32]. Although these studies demonstrated the importance of a restricted conformation, they did not give insights on the preferred conformations required for these peptides in order to bind to one integrin receptor or another. 

**Fig. (1):**
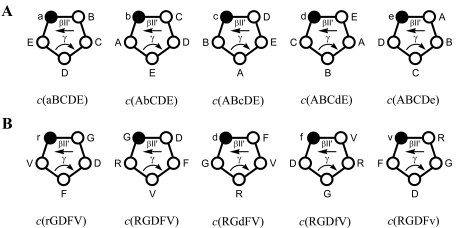
(**A**) Spatial screening of cyclic pentapeptides. The D-amino acid (represented with lower case letters and black dots) tends to occupy the *i*+1 position in the βII’ turn. Therefore different conformations of a bioactive sequence (e.g. ABCDE) can be analyzed without changing the chemical entity of the side chains. (**B**) Spatial screening of RGDFV cyclic pentapeptides. The lead sequence was fixed in different conformations by variation of the chirality of selected residues.

To investigate if and to what extend the spatial orientation of the crucial side chains is involved in activity and specificity of the ligands, we explored the conformational space of cyclic pentapeptides and hexapeptides containing the RGD sequence [33, 34]. Cyclic pentapeptides containing one d- and four l-amino acids prefer a conformation with a βII’ turn, in which the d-residue is located at the *i*+1 position. A loop on the other site of the cycle often involves a γ turn. Therefore, the substitution of each l-amino acid by a d-amino acid will force the adoption of a βII’ turn at different sites of the peptide, allowing the study of various conformations without modifying the chemical nature of the side chains (Fig. **1A**). This concept was first described in 1986 for thymopoietin cyclic pentapeptides analogues [35] and later named “spatial screening” [36, 37].

Based on this approach we synthesized a series of cyclic pentapeptides with the sequence RGDFV (F and V are naturally occurring amino acids next to the RGD sequence in Vn and Fg ECM proteins and indeed showed to be important for the biological activity in an earlier study) [38] and studied the effect on the conformation and the biological activity caused by a single d-amino acid substitution (Fig. **1B**) [33]. As shown in Table **1**, the peptide containing a d-Phe, *c*(RGDfV), showed an increased inhibition of A375 cell adhesion to laminin P1 (20-fold) and to Vn (100-fold) when compared to the control linear peptide GRGDS [20]. The use of d-Val exhibited also improved inhibitory activity but only for the laminin P1 substrate. On the contrary, the insertion of D-Asp or D-Arg had a detrimental effect on activity. The low activity displayed by RGDFv confirmed that the increase in activity for some peptides was due to the spatial orientation of the side chains rather than the presence of a d-residue. Both A375 and HBL-100 cell lines express the αvβ3 integrin, thus inhibition of their binding to Vn indicated an antagonistic effect of the cyclic peptides towards this receptor. Interestingly, *c*(RGDfV) failed to reproduce the same inhibitory effect on αIIbβ3 binding to Fg, demonstrating that the high activity obtained for Vn and laminin P1 substrates was specific for the receptor(s) recognizing these substrates (i.e. αvβ3 and probably αvβ5). 

**Fig. (2):**
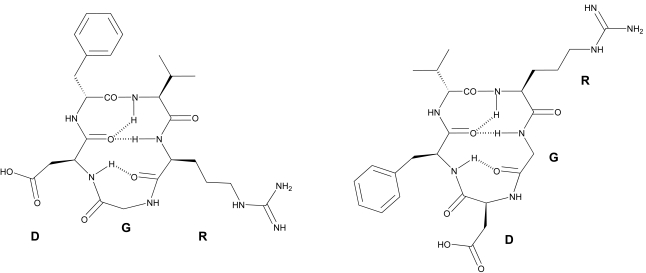
Chemical structure of the cyclic pentapeptides *c*(RGDfV) (left) and c(RGDFv) (right). Dashed lines represent essential hydrogen bonds required to stabilize the βII’ and γ turns.

The conformations of *c*(RGDfV) and *c*(RGDFv) were investigated by NMR spectroscopy combined with molecular dynamic (MD) simulations. Both peptides showed an all-*trans* conformation of all peptide bonds and the expected βII’ and γ turns, with the D-residue at the *i*+1 position (Fig. **2**). The main difference was the position of the RGD motif relative to the cycle turns. In *c*(RGDfV), the RGD motif forms a tight γ turn with Gly in the central position. The Arg and Asp side chains are oriented almost parallel to one another on the same side of the ring. In contrast, in *c*(RGDFv) the Arg and Asp side chains point in opposite directions, keeping the guanidino and carboxy functional groups separated by a larger distance. Comparing these observations with the biological data (Table **1**) we hypothesized that *c*(RGDFv) exhibited the bioactive conformation for the laminin P1 receptor, whereas the distinct conformation of *c*(RGDfV) would be related to the receptor-bound conformation of the Vn receptor. MD simulations further showed that the d-Phe peptide is able to adopt the d-Val peptide’s structure via a βIγ_i_ turn intermediate. However, the opposite conformational transition is not observed for the d-Val peptide, explaining its low activity for Vn and thus selectivity [39]. Concerning the other inactive peptides, the shifts in the position of the RGD sequence within the βII’γ turns for *c*(rGDFV) and *c*(RGdFV) would explain the poor activity of these molecules. The low activity of the cyclic d-Arg analogue was of particular interest, since the substitution of l-Arg by d-Arg in the linear reference peptide GRGDSP did not represent a significant loss of inhibitory activity of cell adhesion to Vn or Fn [31]. Therefore, the decrease in biological potency observed for *c*(rGDFV) was a pure conformational effect on biological activity.

In addition to these studies, a similar spatial screening was performed for cyclic hexapeptides of the sequence *c*(RGDFVA) [33]. Conformational analysis of *c*(RGDfVA) revealed a βII’ turn with D-Phe at the *i*+1 position, but a βII turn (Arg *i*+1, Gly *i*+2) instead of the γ turn. This peptide had a 3 to 5-fold lower inhibitory activity for laminin adhesion when compared to the linear GRGDS, demonstrating that a stretched conformation for cyclic RGD peptides was detrimental for αvβ3-binding. 

Further studies in this direction were pursued by synthesizing libraries of cyclic penta- and hexapeptides where conformational control was introduced by a d-residue and/or proline (turn-inducing amino acids). Detailed conformational analysis of these peptides and their correlation with adhesion inhibitory capacity, allowed a comprehensive description of structure-activity relationships for RGD-peptides binding to integrin receptors [34, 40]. It was corroborated that selectivity of integrin binding peptides strongly depends on the conformation they adopt. For instance, in the *c*(RGDfV) favored conformation, the RGD motif forms a kink around Gly. This conformation seems to be optimal for αvβ3 binding and selective towards αIIbβ3, since *c*(RGDfV) fails to inhibit the binding of this integrin with Fg. On the other hand, some cyclic hexapeptides with no affinity for αvβ3 proved to be highly active inhibitors of the αIIbβ3-mediated platelet aggregation [40]. A closer look on the structural data reveals that αIIbβ3 has a wider RGD binding site (the distance between the C_β_ atoms of Arg and Asp in cyclic hexapeptides is in the range of 0.75-0.85 nm) compared to a narrower binding site in integrins αvβ3 or α5β1, in which cyclic pentapeptides would be better accommodated (C_β_ distances between Arg and Asp below 0.67 nm) [40, 41]. 

**Fig. (3):**
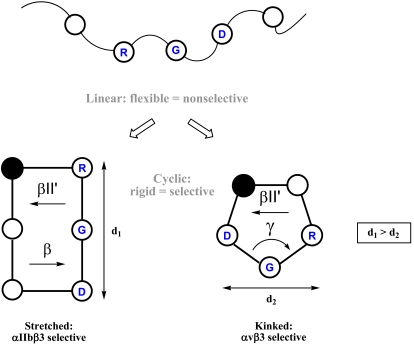
Starting from a linear, conformationally flexible and nonselective peptide, conformational restriction by cyclization and spatial screening leads to rigid and selective structures. The distance between Arg and Asp side chains is represented as *d*. This distance is smaller when the RGD motif adopts a kinked conformation. Adapted from [10].

The above mentioned studies described the first example of a highly active and selective RGD-peptide and established the structural basis to discriminate between different integrin subtypes (Fig. **3**). 

The antagonistic activity of the lead structure *c*(RGDfV) for αvβ3 and its selectivity against αIIbβ3 was also evaluated in a series of studies using the isolated integrin receptors [42]. As shown in Table **2**, the cyclic peptide has more than two orders of magnitude higher potency inhibiting Vn binding to the isolated receptor than the linear peptide. In addition, it shows 350 times lower affinity for the platelet receptor, confirming its selectivity. Antagonistic properties for this peptide towards αvβ3 were further investigated *in vivo*, where a single injection of *c*(RGDfV) disrupted tumor-induced angiogenesis in a chick chorioallantoic membrane (CAM) model [43]. 

Further structure-activity relationship studies of *c*(RGDfV) also explored substitutions at positions 4 (d-Phe) and 5 (Val). Interestingly, the presence of d-Phe and the proton of the amide bond between residues 3 and 4 (Asp and d-Phe) are essential for the activity. In contrast, the amino acid in position 5 has no effect on the biological activity [42]. This finding was of great value for the design of integrin ligands for a number of biomedical applications. E.g., replacement of valine by lysine or glutamic acid retains the integrin binding activity of the peptide providing a new functional group that can be further functionalized. For instance, the cyclic peptide *c*(RGDfK) has been widely used for coating of biomaterials to enhance cell adhesion or as imaging agent for tumor therapy. However, it is not the purpose of this review to focus on these applications, which have been carefully reviewed elsewhere [10, 44-46].

**Fig. (4):**
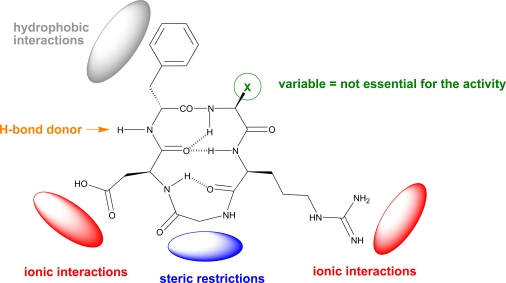
The tripeptide sequence Arg-Gly-Asp is essential for the activity and does not allow any other amino acid combination. Arg and Asp residues might promote ionic interactions with the receptor (with the carboxylate of Asp coordinating divalent cations) whereas the Gly imposes steric restrictions. Position 4 requires a hydrophobic residue in the D-configuration (i.e. D-Phe) for optimal side chain orientation and interaction with the receptor. The amide bond between residues 3 and 4 also participates in the binding and therefore may act as a hydrogen bond donor. Finally, position 5 can accommodate a number of residues without an impact in the biological activity.

These findings served to propose a receptor model for optimal binding with the αvβ3 integrin (Fig. **4**). This model highlights the main pharmacophoric features of *c*(RGDfV) for the interaction with the αvβ3 receptor [41, 42]. 

The lead structure *c*(RGDfV) was subjected to a number of modifications such as the substitution of peptide bonds with thioamides [47] or their reduction [48], the incorporation of turn mimetics [49], the use of sugar amino acids [50] and the synthesis of retro-inverso analogues [51]. Together with these strategies the design and synthesis of totally non peptidic antagonists was also approached [9, 52]. However, the most important structural modification turned out to be the incorporation of *N*-methyl amino acids into the peptide sequence [16]. This approach led to the discovery of Cilengitide, the first anti-angiogenic drug targeting integrins. 

### 1.3 *N*-Methyl Scan of the Lead Structure *c*(RGDfV)

*N*-Methylation of peptide bonds has proven to be a powerful technique for medicinal chemists to increase the potential of peptides as drugs [53]. *N*-Methylation is currently used to improve the biological activity and selectivity profile of peptides [54] and also to overcome their pharmacokinetic limitations i.e. increasing their metabolic stability and bioavailability [55, 56]. This strategy is also a valuable tool to explore the bioactive conformation of biologically relevant peptides, since the introduction of *N*-methyl groups promotes conformational constraints that may enhance the population of single conformers essential for the biological activity [57, 58].

Most of these unique properties were already known in the 90s. *N*-Methylation was in particular used for conformational studies [59, 60], and to improve the peptide’s pharmacokinetic properties [61] and receptor selectivity [62, 63]. In an interesting study, DeGrado and coworkers showed that *N*-methylation of the Arg residue in a class of RGD cyclic peptides improved their antagonistic activity for αIIbβ3 due to conformational constraints in the peptide’s structure [64]. In a further study, they also proved how the exchange of an *N-*methylated d,l-configurated dipeptide motif by a l,l-dipeptide unit in a cyclic RGD peptide, resulted in a change of selectivity from an αIIbβ3-selective ligand to an αvβ3-selective peptide [65, 66]. This shift in selectivity is based on the distinct distances between the C_β_ atoms of Arg and Asp residues, which are much smaller for the ligands selective for αvβ3. These results supported our previous hypothesis [40], which were finally confirmed through X-ray structure analysis of the αIIbβ3 receptor [13] (see section 2.1).

These findings together with the positive effects in activity and selectivity described for the *N*-methylation of other bioactive peptides, inspired us to perform an *N*-methyl scan of the lead peptide structure *c*(RGDfV) [16]. The resulting five *N*-methylated peptides are shown in Fig. (**5**). 

The ability of these analogues to inhibit the binding of Vn and Fg to immobilized αvβ3 and αIIbβ3 receptors was compared with the original cyclic peptide *c*(RGDfV) and the linear control GRGDSPK (Table **3**).

Regarding the antagonistic activity for αvβ3, the analogues with Asp or d-Phe residues *N*-methylated showed a lower activity than the control peptides. Remarkably, peptide **4** is one order of magnitude less active compared to the linear peptide. In addition to conformational reasons, this behavior could be attributed to the loss of a hydrogen bond donor in the peptide bond between Asp and D-Phe due to *N*-methylation, since this amide bond was reported to contribute to the activity [42]. The modification of the Gly residue (e.g. substitution by alanine or β-alanine) leads to suppression of activity in RGD ligands, therefore the rather high (45 nM) biological activity observed for compound **2** was unexpected. From this series only analogue **5** displayed an enhanced activity compared to *c*(RGDfV). This peptide, which contains *N*-methyl Val, had an antagonistic affinity of 0.58 nM for αvβ3 and showed only low activity for αIIbβ3 (0.86 µM) being 1500 times more selective in inhibiting the binding of Vn to αvβ3 than Fg to αIIbβ3. Interestingly, it also displayed a relatively high affinity for αvβ5 (in the nanomolar range). At this time, the role of this integrin was not known, hence a biselective compound was considered of great interest. This highly active and selective compound was chosen for drug development by Merck and later named Cilengitide. 

**Fig. (6):**
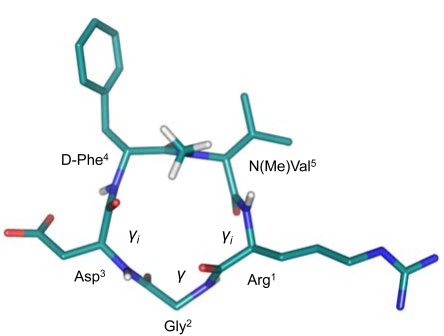
Three-dimensional structure of Cilengitide obtained by NMR and MD calculations. For clarity only the protons from the *N*-methyl group and the amide bonds are shown. The different turns observed are indicated.

In order to study the effect of *N*-methylation in the conformation of the peptide, the three-dimensional structure of Cilengitide was determined. Significant structural differences were found. Due to steric repulsion the amide bonds between Asp^3^-d-Phe^4^ and Val^5^-Arg^1^ are placed in a more perpendicular orientation regarding the plane of the peptide backbone, and two inverse γ turns (γ_i_) are observed with Arg^1^ and Asp^3^ at the *i*+1 position. A γ turn is also observed with Gly^2^ in position *i*+1 (Fig. **6**). The γ_i _turns compensate the hydrogen bond that should be observed in a* β*II’turn, which is no longer present. The rotation of the amide bonds Asp^3^-d-Phe^4^ and Val^5^-Arg^1^ also influences on the orientation of the Asp and Arg side chains which move towards a more pseudoequatorial orientation. On the contrary, the non-methylated peptide *c*(RGDfV) places these side chains in a pseudoaxial conformation. This finding suggests that although a kink in the RGD motif is necessary for αvβ3 activity and selectivity towards αIIbβ3, it is not as essential for the binding affinity as previously postulated (a distance between Arg and Asp C_β_ atoms below 0.67 nm) [40]. Remarkably, the conformation determined in solution turned out to be identical to the one described for Cilengitide bound to the αvβ3 integrin (see section 2.1).

To summarize this first part, we have described the design and synthesis of Cilengitide as a potent and selective integrin ligand. A major milestone in the design of this potent compound was the introduction of d-amino acids to explore the optimal spatial conformation required for biological activity of cyclic RGD-containing peptides. Our findings improved drastically the affinity of linear RGD peptides for integrin binding, and also established the structural basis necessary for integrin selectivity. In the next chapter, we explore the role of integrins in angiogenesis and cancer, and the biochemical background of Cilengitide’s biological activity. 

## 2 BIOCHEMICAL BACKGROUND

### 2.1 Integrins in Angiogenesis and Tumor Vasculature

Cell attachment and detachment is crucial for function of all higher organisms. Integrins provide controlled adhesion to different tissues and signaling into the cell in case of proper adhesion. One of the most important processes in embryogenesis, wound healing and female menstrual cycle is angiogenesis, since the transport of nutrients and oxygen throughout the body to organs and tissues is indispensible for the organism [4, 67]. Integrins have distinct roles and are critical mediators and regulators in the physiological and pathological angiogenesis, including tumor angiogenesis, by activating kinases [68].

**Fig. (7):**
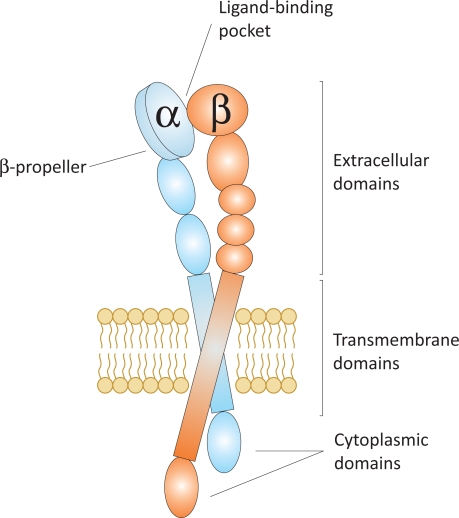
Schematic representation of an integrin in the unligated state.

Integrins are non-covalently associated heterodimers of one α and one β subunit, altogether forming more than 24 integrins using 18 α and 8 β subunits [7]. The α and β subunits are both type-I membrane proteins with a large extracellular domain and a generally short, non-catalytic cytoplasmic tail, linked by a single transmembrane region (Fig. **7**) [69]. The physical interaction of integrins with ECM proteins promotes cell adhesion and migration, and affects signaling pathways that regulate cell proliferation, survival, and differentiation as well as cytoskeletal organization and force generation [70].

Signaling by integrins can induce or prevent apoptosis as they regulate both the expression and activity of pro-apoptotic proteins. Additionally, integrins play an important role in the molecular regulation of lymphangiogenesis, which has been recently reviewed in the literature [6, 71]. Integrins also contribute to the regulation of immunity, inflammation and hemostasis and are involved in many pathological conditions such as cancer, autoimmune diseases or atherothrombosis [1, 72]. Although numerous *in vivo *and *in vitro* experiments have shown that integrins expressed on endothelial cells play an important role in cell growth, survival and migration during angiogenesis [6] and apoptosis, their exact mode of action and mechanisms remain unclear [73]. However, many cancer cells overexpress certain integrins to control migration, extravasation, and homing [70].

The integrins involved in angiogenesis comprise the heterodimers α1β1, α2β1, α4β1, α5β1, α6β1, α6β4, α9β1, αvβ3, αvβ5 and the glial cell integrin αvβ8 [6]. These receptors are targets of both angiogenic activators and inhibitors. Some integrins, such as α5β1, prefer a single ligand (i.e. Fn), whereas other integrins can bind to distinct ECM proteins [8]. This is the case for instance of αvβ3 which binds to Vn, Fn and Fg among others. The combination of the integrins expressed on a given cell dictates to what extent the cell will adhere to and migrate on different matrices [7]. The binding of integrins to their natural ligands is in nearly half of the over 20 known integrins mediated by the RGD recognition motif (see section 1.1). RGD-recognizing integrins include α5β1 and all the types of αv integrins.

**Fig. (8):**
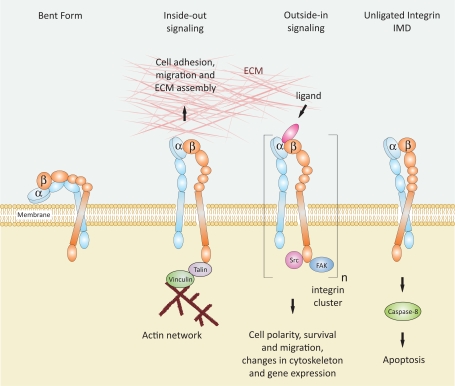
Schematic representation of integrin activation states and signaling mechanisms. In the bent form the integrin head group points inwards towards the cell surface and has low affinity for ligands [80]. During “inside-out signaling” an intracellular activator binds to the β-subunit, induces a conformational change leading to increased affinity for extracellular ligands [72]. This process is known to regulate cell adhesion, migration and invasion. During “outside-in signaling” a ligand binds to the integrin and can induce, because of multivalency, integrin clustering. Activation of a signal cascade leads to intracellular signals, which regulate cell polarity, survival and migration, changes in cytoskeleton and gene expression. The presence of unligated integrins can activate caspase-8, and as a consequence, induce apoptosis in a process known as IMD [78, 79].

Integrin binding to ligands in the ECM induces conformational changes in the integrin’s structure and contributes to clustering of heterodimers into oligomers [72]. This leads to intracellular signals through multiple activation of signaling proteins. This process is known as “outside-in signaling” and controls cell polarity, cytoskeletal structure, gene expression and cell survival. Integrins are bidirectional signaling machines, and they can also respond to intracellular signals, “inside-out signaling”, which regulate the adhesiveness to the ECM ligands and thus cell invasion and migration [67]. Detached cells undergo apoptosis resulting from a variety of events [74]. Thus, the prevention of integrin mediated adhesion to the ECM leads to apoptosis and suppression of invasive events like liver metastasis and angiogenesis [43]. In addition, detachment of cells from the surrounding ECM and appearance of unligated integrins was demonstrated to trigger the activation of caspase-8 and consequently, apoptosis [75-77], a mechanism called “integrin-mediated death” (IMD) (Fig. **8**) [78, 79].

The first crystal structure of the extracellular segment of the αvβ3 integrin published in 2001 was a major breakthrough [11]. It revealed that the *N*-terminal segments of both the α and β subunits assemble in an ovoid-like head from which two nearly parallel tails emerge. The αv tail is composed of three β-sandwich domains: an Ig-like thigh domain and two very similar domains that form the calf module. The β3 subunit consists of a plexin-semaphorin-integrin module which is found in several protein families (plexins, semaphorins and integrins), four epidermal growth factor (EGF) domains and a β-tail domain. The ligand binding site of the RGD ligand is located at the interface of the so called β-propeller domain formed from αv and a βA domain from β3.

**Fig. (9):**
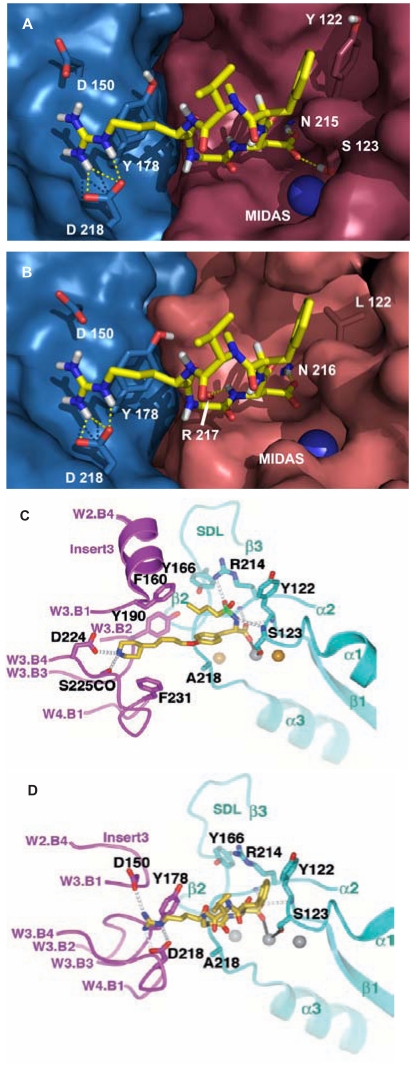
Cilengitide bound to αvβ3 (**A**) and to αvβ5 (**B**) [82]. Binding of Tirofiban to αIIbβ3 (**C**) and of Cilengitide to αvβ3 (**D**) [13]. Figures C and D are obtained, with permission, from Nature Publishing group, Ref 13 (2004), Macmillan Publishers Ltd. All rights reserved.

In 2002 the crystal structure of the extracellular segment of the integrin αvβ3 complexed with Cilengitide in the presence or absence of the pro-adhesive cation Mn^2+^ was elucidated [12]. The structure of Cilengitide and αvβ3 in the presence of Mn^2+^ revealed that the peptide inserts into a crevice between the β-propeller and the βA domain on the integrin head. Cilengitide forms a slightly distorted pentagon (see also Fig. **6**) during the interaction, with the arginine and the aspartic acid side chains of the RGD motif pointing in opposite directions. The guanidinium group of Cilengitide is fixed inside a narrow groove (Fig. **9A**) formed by the D3-A3 and D4-A4 loops of the β-propeller by a bidentate salt bridge to Asp^218^ and another salt bridge with Asp^150^. The carboxylate group of Cilengitide points into a cleft between two loops of the βA domain, coordinating a Mn^2+^ ion at the metal ion-dependent adhesion site (MIDAS). It is additionally involved in hydrogen bonds with the backbone amides of Tyr^122^ and Asn^215^. The glycine lies at the interface between the α and β subunits and makes several hydrophobic interactions with the integrin surface, including a contact with the carbonyl oxygen of Arg^216^. As previously mentioned, the conformation of Cilengitide bound to αvβ3 is almost identical to the conformation of Cilengitide in aqueous solution determined by NMR spectroscopy [16]. These findings support our previous observations, based on ligand-oriented structure-activity studies in the process of the development of Cilengitide, and are in agreement with the fact that the RGD sequence is crucial for optimal αvβ3 binding and that these pharmacophoric groups cannot be replaced. The remaining two residues of Cilengitide face away from the αβ interface and thus a large fraction of the cyclic peptide has no contact with the αvβ3 integrin surface.

A three-dimensional model for the human αvβ5 integrin was obtained using homology modeling based on the experimental three dimensional structure of αvβ3 in its bound conformation [81]. The work assumed that the αv and the β5 subunit assemble in a similar manner as found for αvβ3 and thus Cilengitide interacts with both integrins in a related way (Fig. **9B**). The homology model for α5β1 was also reported [82]. These models paved the way for the rational design of antagonists of these integrins [83].

The publication of the co-crystal structure of Tirofiban associated with αIIbβ3 in 2004 elucidated the difference between the binding pockets of αIIbβ3 and αvβ3 (Fig. **9C**, **9D**) [13]. The observation that the selectivity for αIIbβ3 and αvβ3 is conferred by the distance between the acidic and the basic moiety of the respective ligand [41], has now been structurally confirmed. The Asp^224^, involved in the hydrogen bond with the basic ligand-mimetic side chain, is located in the deeper β-propeller pocket of αIIb, whereas the residues Asp^150^ and Asp^218^ of the αvβ-propeller are closer to its shallower pocket.

### 2.2 The Role of αvβ3, αvβ5 and α5β1 in Cancer

The repertoire of integrins in endothelial cells of angiogenic vessels differs from the integrins expressed in resting endothelial cells [84]. Both integrins αvβ3 and αvβ5 are expressed in various cell types such as endothelial cells, fibroblasts, epithelial cells, osteoblasts, and smooth muscle cells and are upregulated in endothelial cells undergoing angiogenesis. Additionally, they are highly upregulated on endothelium during tumor angiogenesis.

Tumors with only a small volume of a few cubic millimeters may rest for months or years without neovascularization, when the balance between pro- and anti-angiogenic signals does not favor the growth of the local vasculature [85, 86]. Initiated by local hypoxia, tumor cells can switch to the angiogenic phenotype activating the expression of vascular endothelial growth factors (VEGFs), which can recruit and subsequently activate a family of tyrosine kinase receptors [84, 87]. This early event in tumor progression called the “angiogenic switch” enables tumors to attract new blood vessels to establish a vascular connection with the host and support the growth of both, the angiogenic and the non-angiogenic cells [85]. Inhibition of VEGF signaling therefore is one of the most prominent targets for anti-angiogenic drugs, as growth factor signaling leads to expression of integrins which allow for example tumor cells and endothelial cells to migrate to the stimulus [88].

During the invasive phase endothelial cells penetrate the underlying basement membrane, proliferate and migrate on the ECM. This process enables the cancer cell to pull itself forward into the tissue and arranges the endothelial cells into functional vessels [70]. In this manner, angiogenesis helps tumor cells to gain access to the circulation as well as provides nutrients and oxygen to cancer cells. As key components in the interaction between activated, proliferating endothelial cells and the surrounding stroma, integrins are essential in cancer metastasis and tumor progression. They regulate tumor cell survival – by preventing pro-apoptotic signals – and malignancy in the ligated and the unligated state [7].

The first evidence for the involvement of specific integrins in pathological angiogenesis was found in studies employing antibodies and small molecules directed against the αvβ3 integrin [43, 89]. The αvβ3 and αvβ5 integrins are usually expressed at low levels in most adult epithelia but can be highly upregulated in some tumors. They are not only highly expressed on morphologically abnormal tumor vasculature, but also on tumor cells, including gliomas [6, 90]. Positron emission tomography (PET) using [^18^F]Galacto-RGD [91] and validation by immunohistochemistry revealed αvβ3 expression in different solid tumors of patients but lack of expression in normal tissues (e.g. benign lymph nodes, muscles) [92]. It has been shown that activation of αvβ3 is required for metastasis in a breast cancer carcinoma model [93] and that expression of αvβ3 and αvβ5 in tumor vasculature correlates with the malignancy of neuroblastoma [94, 95]. The selective upregulation of the αvβ3 receptor in malignant glioma suggests a major role for this integrin in this type of cancer. However, it is well documented, that not only αvβ3, but also other integrins, such as αvβ5 integrin, are upregulated in this cancer [96, 97]. 

Despite the reported important role of αvβ3 in angiogenesis, mice lacking the αv gene show extensive vasculogenesis and angiogenesis [98]. Additionally, mice lacking β3 and β5 integrins display enhanced pathological angiogenesis and accelerated tumor growth [99]. The dispensable role of αvβ3 in developmental angiogenesis is consistent with the finding that humans who carry a null mutation in the β3 subunit (Glanzmann thrombasthenia) exhibit normal vascular development and angiogenesis [100, 101]. Due to the discrepancy between these studies and the observation that αvβ3 inhibitors suppress angiogenesis, the question of whether the αvβ3 integrin regulates angiogenesis in a positive or rather negative way is still under debate [7, 73, 78, 102].

The α5β1 integrin and its ligand, Fn, are known to be pro-angiogenic [103]. Indeed α5β1 is selectively expressed in angiogenic vasculature. Similarly to αvβ3 and αvβ5, α5β1 is also highly expressed in endothelium during tumor angiogenesis both in mice and in humans, but poorly expressed on normal quiescent blood vessels. α5β1 promotes epithelial cell survival and induces angiogenesis *in vitro*, whereas genetic ablation results in embryonic lethality with disruption in blood vessel formation [104, 105] and blocking antibodies or peptides have shown to inhibit angiogenesis *in vivo* [103].

The αvβ3, αvβ5 and α5β1 integrins have partially overlapping ligand specifities and α5β1 might be able to substitute αvβ3 or αvβ5 biological functions. This could be a reasonable explanation for the discrepancy above mentioned [84]. The αvβ3-dependent pro-angiogenic pathway is different from that regulated by αvβ5. Integrin blocking experiments have shown that VEGF-induced angiogenesis is dependent on αvβ5, whereas angiogenesis induced by basic fibroblast growth factor (bFGF) expresses αvβ3 [106]. The expression of the integrin α5β1 is induced by a variety of angiogenic stimuli, such as bFGF and others, but not by VEGF [103, 107].

### 2.3 Cilengitide as Integrin Antagonist

Due to their primary expression on activated endothelial cells, the integrins αvβ3, αvβ5, and α5β1 are attractive targets for cancer therapy [108] and the treatment of non-malignant angiogenic disorders [109]. Especially in the case of solid tumors, anti-angiogenic molecules represent a new potent concept of therapy. The inhibition of integrin-ligand interactions suppresses cellular growth and induces apoptotic cell death [43, 110, 111]. The vast number of reported integrin antagonists comprises monoclonal antibodies, peptide and peptidomimetic antagonists and small molecules [10, 112].

Therapies directed against angiogenic blood vessels take advantage of the distinct biochemical properties of neovascular vessels versus resting vasculature. Currently, several compounds targeting integrins are in clinical trials as potential drugs for the treatment of numerous diseases including cancer [6]. Among them, Cilengitide is the first integrin antagonist in clinical phase III for treatment of glioblastoma and in phase II for several other tumors. This drug is the only anti-angiogenic small molecule showing subnanomolar antagonistic activity for αvβ3 and affinities in the low nanomolar range for αvβ5 and α5β1. 

Cilengitide acts as a highly potent inhibitor of angiogenesis and induces apoptosis of growing endothelial cells *via *the inhibition of the interaction between integrins with their ECM ligands [113, 114]. Cilengitide was shown to influence cellular adhesion to αvβ3 ligands, to induce increased apoptosis after detachement of αvβ3 and αvβ5 expressing cells *in vitro* [113] and to block the growth of human xenografts in nude mice [115]. Additionally, it revealed anti-angiogenic and anti-tumor activity in various animal models [116-118]. The inhibition of αv integrins resulted in significant reduction of functional vessel density and retardation of tumor growth and metastasis *in vivo* [117]. 

Recently it has been shown, that αvβ5 mediates metastasis and that treatment with Cilengitide of tumor cells which express the αvβ5 integrin but not the αvβ3 integrin, effectively prevented metastasis formation [119]. It is documented, that Cilengitide induces apoptosis in αv expressing tumor cell lines by detaching them from Vn and tascin, matrix proteins known to be essential for tumor growth and invasion and it also induces apoptosis in both brain capillary and brain tumor cells [113]. Additionally, it has been documented that treatment with Cilengitide decreases osteolysis of breast cancer metastasis in nude rats and the volume of the soft tissue tumor components [120]. A study performed in 2009 proved that hypoxia stimulates the αvβ3 and αvβ5 integrin pathways through focal adhesion kinase (FAK) and that hypoxia activates FAK in gioblastoma cell lines [121]. Treatment of glioblastoma cells with Cilengitide led to a significant and dose dependent decrease of hypoxia-inducible factor 1α (HIF-1 α) intracellular level under hypoxic conditions. This study suggests that αvβ3 and αvβ5 are activated by hypoxia and are key regulators of glioma response to hypoxic conditions by controlling HIF-1 α degradation.

Finally, it has been shown that low nanomolar concentrations of Cilengitide paradoxically stimulate tumor growth *in vivo* by promoting VEGF-mediated angiogenesis [122], an observation that has been a matter of debate in the literature [123, 124]. This might be the influence of the ligand in the first step of the multistep mechanism to activate integrins and form focal contacts and finally focal adhesion [72]. As signal transduction requires dissociation of the transmembrane helices and aggregation, blocking the multivalent binding at higher Cilengitide concentrations might cause the anti-angiogenic effect. However, the concentrations used in clinical trials largely exceed the described “pro-angiogenic” concentration of Cilengitide, and therefore, such a biological effect is not expected in clinical application [123] (see section 3).

As reported, Cilengitide demonstrated anti-angiogenic and anti-tumor qualities and inhibition of tumor metastasis in many preclinical studies. Additionally, integrin antagonists seem to synergize with already established therapeutic treatments, such as radiotherapy (RT) and chemotherapy [17]. Stabilizing effects in highly vascularized solid tumors by Cilengitide in combination with chemotherapeutic drugs were demonstrated [117, 125]. Furthermore, Cilengitide showed increased efficacy of RT in endothelial cells and non-small cell lung cancer (NSCLC) [126]. The combination of an integrin antagonist and RT showed a significant delay of tumor growth in glioblastoma xenografts compared with either treatment individually [127]. Irradiation of tumors reduces the local tumor growth, but at the same time upregulates αvβ3 expression [127] and enhances local invasion and metastatic spreading [17]. Therefore, it is plausible that Cilengitide as an integrin antagonist may normalize the tumor vasculature and attenuate some of these radiation-induced effects.

## 3 CILENGITIDE IN THE CLINICS

The above mentioned preclinical studies showed a promising synergy between Cilengitide and radio-chemotherapy in order to normalize tumor vasculature and attenuate tumor invasion and metastases. In a key preclinical study made by MacDonald and collaborators, treatment with Cilengitide showed reduction of brain tumor and increased survival on mice with orthotopic brain tumors compared to mice treated with an inactive peptide. Interestingly, when the tumors were grown in the subcutis of nude mice (heterotopic model), no inhibition of tumor growth was observed for the mice treated with Cilengitide [116]. These findings suggested that brain tumors, which are highly angiogenic, were more susceptible to growth inhibition by integrin antagonists and led to subsequent clinical investigation. A summary of the most representative clinical trials completed of Cilengitide in brain tumors (recently reviewed in [17]) and other cancer types can be seen in Table **4**. 

### 3.1 Cilengitide as Single Agent for Glioblastoma Treatment

The first report of biological activity by an integrin antagonist was documented for Cilengitide in a phase I trial in patients with recurrent malignant glioblastoma (GBM) (Table **4**) [128]. The study was undertaken to determine the toxicities and maximum-tolerated dose (MTD) of Cilengitide in patients with malignant primary brain tumors. In this study Cilengitide showed an unexpected single agent (administered without any other drugs) activity for these tumors with limited toxicity to doses up to 2,400 mg/m^2^. Out of the 51 patients of the study, five showed objective response (OR): two complete response (CR) with improved functional status and no tumor recurrence and three partial response (PR). The pharmacokinetics (PK) of Cilengitide in this study were comparable to other phase I studies in patients with advanced solid tumors, in which it was shown that Cilengitide has an apparent terminal half-life of 3 to 5 h and can be safely administered on a twice per week infusion schedule [129, 130]. Interestingly, peak plasma concentrations which showed anti-tumor effects in pre-clinical models were obtained at doses ≥ 120 mg/m^2^ [129]. Another phase I study was conducted in children with refractory brain tumors [131]. Dose limiting toxicity (DLT) was not observed but three serious cases of intratumoral hemorrhage (ITH) were documented. However, this study concluded that a 1,800 mg/m^2^ dose of Cilengitide in children with brain tumor was devoid of increased risk of ITH. One patient had CR and six more had stable disease (SD). These studies were promising and encourage pursuing further phase II studies in which the most appropriate dose of Cilengitide or the synergy of this integrin inhibitor with chemotherapy agents and RT were evaluated [132]. A phase II study for patients with recurrent GBM who required tumor resection was set to measure a progression-free survival rate at 6 months (PFS-6) and examine the delivery of Cilengitide into tumor [133]. Treatment with Cilengitide was well tolerated and episodes of post-operative hemorrhages were not observed. Preliminary data showed significantly increased concentrations of the drug in the tumor compared to plasma concentrations, demonstrating a dose-related good delivery of Cilengitide into the brain tumor. A multicenter, open-label, randomized phase II study was conducted to further evaluate the efficacy and safety of Cilengitide among recurrent GBM patients [134]. As previous clinical studies showed responses at both the lower and the higher dose levels, two Cilengitide dose concentrations were evaluated: an intermediate-low (500 mg) dose and an intermediate-high (2000 mg) dose, relative to the previous studies. Cilengitide showed an excellent safety profile and anti-tumor activity at both concentrations, though showing a more favorable trend for patients treated with the higher dose. Radiographic response (RR) (5% vs. 13 %); PFS-6 (10% vs. 15%); and median overall survival (OS) (6.5 months vs. 9.9 months) were obtained comparing 500 mg vs. 2000 mg, respectively. Recently reported follow-up (> 4 years) data showed that long-term survival rates were consistently greater with 2000 mg (10.0% after 54 months) versus 500 mg (2.4% after 54 months) [135].

### 3.2 Cilengitide in Combination with Radio-Chemotherapy for GBM Treatment

Preclinical studies demonstrated that Cilengitide in combination with chemotherapy agents and in particular with RT and chemotherapies could have an enhanced anti-tumor activity [127]. Furthermore, the low toxicity profile observed for Cilengitide suggested that it could be administered safely in combination with cytotoxic therapy. For these reasons, several clinical trials on this direction were conducted. A randomized phase II trial combining Cilengitide with Temozolomide (TMZ) and RT was reported. The objective was to determine safety of this therapy combination and OS in 112 patients with newly diagnosed GBM [136]. Combination of Cilengitide (500 or 2000 mg) with TMZ and RT was well tolerated and showed improved survival (median OS: 18.9 months; OS at 12 months 79.5 % of patients). Positive effects for this therapeutic combination were also recently reported for a similar phase I/IIa study [137]. Interestingly, the authors of this study showed that patients whose tumors had *O*^6^-methylguanine-DNA methyltransferase (MGMT) promoter methylation, were more susceptible to the treatment exhibiting longer PFS and OS. In this sense, methylation of MGMT was already described to be a putative marker for benefit from TMZ in GBM treatment [138]. On the basis of these results an international, randomized, controlled phase III trial (CENTRIC) was launched in 2008. This trial was organized by Merck KGaA (Germany) in collaboration with the European Organisation for Research and Treatment of Cancer (EORTC) and the Canadian Brain Tumor Consortium (CBTC) [139]. The study is currently recruiting participants, with an estimated enrollment number of 504 patients. The study completion is estimated on June 2016, with primary outcome measures on September 2012 [140]. Only GBM patients with MGMT promoter methylation will be considered for this study, and Cilengitide will be administered at a unique i.v. high dose of 2000 mg twice weekly in combination with TMZ/RT. If progression is not observed, Cilengitide treatment will be continued for up to 18 months. In parallel, a randomized phase II clinical trial has been designed for patients with GBM showing no methylation on MGMT gene’s promoter (CORE study) [141], where a recruitment of 264 patients is estimated [140]. Enrollment of children and young adults (6 months to 21 years) with newly diagnosed diffuse intrinsic pontine glioma in a phase I study has also recently started (CILENT-0902, July 2010) [140]. This study will determine the safety and pharmacokinetics of Cilengitide with RT. A summary of these, and other studies currently in progress in patients with GBM are listed in Table **5**.

### 3.3 Cilengitide in Other Cancer Types 

Cilengitide has also been tested for other cancer types different than GBM with mixed results. For instance, the use of Cilengitide in combination with Gemcitabine in a phase II trial in advanced unresectable pancreatic cancer showed no survival benefit compared to treatment with Gemcitabine alone [142]. Currently a number of phase I and II trials with different cancer types are in progress (see Table **5**) [140]. The CERTO study is a multicenter, open-label, randomized, controlled phase II study with a safety run-in part in patients with advanced NSCLC. This study will evaluate both safety and efficacy of Cilengitide treatment in combination with Cetuximab, and platinum-based chemotherapy (Cisplatin/Vinorelbine or Cisplatin/Gemcitabine) [140, 143]. Patients with locally advanced NSCLC are also being recruited for a phase I study with a combination of RT and chemotherapy (Cisplatin and Vinorelbine) with Cilengitide [140]. Cilengitide added to Cisplatin, 5-fluorouracil (5-FU), and Cetuximab is also being evaluated in an open-label, randomized, controlled phase I/II study (ADVANTAGE) in subjects with recurrent and/or metastatic squamous cell carcinoma of the head and neck (SCCHN) [140, 143]. Other evaluations of Cilengitide in, metastatic or not, prostate cancer [140, 144, 145] and brain metastases from lung cancer are also in progress (see Table **5**).

## CONCLUSIONS

The discovery 30 years ago of the RGD motif in Fn was a major breakthrough in science. This tripeptide sequence was also identified in other ECM proteins and was soon described as the most prominent recognition motif involved in cell adhesion. Extensive research in this direction allowed the description of a number of bidirectional proteins, the integrins, which were able to recognize and bind to the RGD sequence. Integrins are key players in the biological function of most cells and therefore the inhibition of RGD-mediated integrin-ECM interactions became an attractive target for the scientific community.

However, the lack of selectivity of linear RGD peptides represented a major pitfall which precluded any clinical application of RGD-based inhibitors. The control of the molecule’s conformation by cyclization and further spatial screening overcame these limitations, showing that it is possible to obtain privileged bioactive structures, which enhance the biological activity of linear peptides and significantly improve their receptor selectivity. Steric control imposed in RGD peptides together with their biological evaluation and extensive structural studies yielded the cyclic peptide *c*(RGDfV), the first small selective anti-angiogenic molecule described. *N*-Methylation of this cyclic peptide yielded the much potent *c*(RGDf(*N*Me)V), nowadays known as Cilengitide.

The fact that brain tumors, which are highly angiogenic, are more susceptible to the treatment with integrin antagonists, and the positive synergy observed for Cilengitide in combination with radio-chemotherapy in preclinical studies, encouraged subsequent clinical trials. Cilengitide is currently in phase III for GBM patients and in phase II for other types of cancers, with to date a promising therapeutic outcome. In addition, the absence of significant toxicity and excellent tolerance of this drug allows its combination with classical therapies such as RT or cytotoxic agents. The controlled phase III study CENTRIC was launched in 2008, with primary outcome measures due on September 2012. The results of this and other clinical studies are expected with great hope and interest. 

## Figures and Tables

**Fig. (5):**
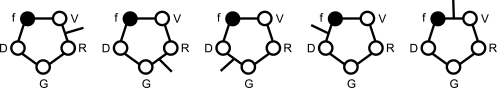
*N*-methylated cyclic pentapeptides derived from *c*(RGDfV).

**Table 1:** Inhibitory Capacity (IC_50_) of RGD-Containing Peptides for Cell Adhesion on Vn or Laminin Fragment P1

Peptide	IC_50_ (µM) A375 Adhesion		IC_50_ (µM) HBL-100 Adhesion	
	P1	Vn	P1	Vn
c(**r**GDFV)	114	>120	25	>120
c(RG**d**FV)	>120	>120	20	>120
c(RGD**f**V)	1.0	0.2	0.1	0.1
c(RGDF**v**)	1.9	20	0.9	30
RGDF**v**	29	82	42	>170
GRGDS	18	15	5	14

For clarity only the more representative peptides and cell lines from the initial study are shown [33].

**Table 2:** Biological Activity (IC_50_) of the αvβ3-Selective Peptide *c*(RGDfV) Compared to Control Linear Peptide GRGDSPK in Inhibiting the Binding of Vn and Fg to Isolated Integrins αvβ3 and αIIbβ3 Respectively

Peptide	IC_50_ (µM) αvβ3	IC_50_ (µM) αIIbβ3	Selectivity αIIbβ3/αvβ3
GRGDSPK	1.2 ± 0.27	5.4 ± 2.0	4.5
*c*(RGDfV)	0.0049 ± 0.0001	1.7 ± 0.38	347

The selectivity for these receptors is expressed as the ratio between the IC_50_ values for each integrin subtype [42].

**Table 3:** Biological Activity (IC_50_) of *N*-Methylated Cyclic Peptides and Standard Peptides in Inhibiting the Binding of Vn and Fg to Isolated Integrins αvβ3 and αIIbβ3, Respectively

Peptide	IC_50_ (µM) αvβ3	IC_50_ (µM) αIIbβ3	Selectivity αIIbβ3/αvβ3
GRGDSPK	0.21	1.7	8.1
*c*(RGDfV)	0.0025	1.7	680
**1**, *c*(-N(Me)R-GDfV)	0.0055	5.2	945
**2**, *c*(R-N(Me)G-DfV)	0.045	> 10	n.c.
**3**, *c*(RG-N(Me)D-fV)	0.56	> 10	n.c.
**4**, *c*(RGD-N(Me)f-V)	1.4	> 10	n.c.
**5**, *c*(RGDf-N(Me)V-)	0.00058	0.86	1483

The selectivity for these receptors is expressed as the ratio between the IC_50_ values for each integrin subtype [16].

**Table 4:** Completed Clinical Trials of Cilengitide in Brain Tumors and Other Types of Cancer

Author/Year	Trial	No. Patients	Purpose	Disease Setting	Cilengitide Dose^a^	Main Results	Ref.

Eskens 2003	Phase I	37 patients	Determine safety, toxicity and PK	Metastatic solid tumors	Single agent 30 to 1600 mg/m^2^	No DLT	[129]
						Half-life: 3 to 5 h	
						No CR or PR. 3 SD	

Friess 2006	Phase II	89 patients	Determine safety, PK and OS	Unresectable pancreatic cancer	Cilengitide (600 mg/m^2^) + Gemcitabine	No clinical differences compared to gemcitabine	[142]
						No survival benefit	

Hariharan 2007	Phase I	20 patients	Determine safety, toxicity and PK	Advanced solid tumors	Single agent 600 or 1200 mg/m^2^	Well tolerated	[130]
						Half-life: 4 h	
						No CR or PR., 4 SD	

Nabors 2007	Phase I	51 patients	Determine MTD Evaluate the use of perfusion MRI in patients with GBM	Recurrent GBM	Single agent 120 to 2400 mg/m^2^	No DLT and MTD	[128]
						No bleeding	
						Tolerated at 2,400 mg/m^2^	
						2 CR, 3 PR and 16 SD	

MacDonald 2008	Phase I	31 patients	Determine MTD and DLT in children with refractory brain tumors	Pediatric brain tumors	Single agent 120 to 2400 mg/m^2^	No DLT and MTD	[131]
						3 cases of ITH	
						1800 mg/m^2^ safe dose	
						1 CR, 6 SD	

Gilbert 2007	Phase II	30 GBM patients	Measure a PFS-6 Examine the delivery of Cilengitide into tumor	GBM requiring tumor resection	Single agent 3 doses (500 or 2,000 mg) before op. After: 2000 mg	Post-op. hemorrhages not observedCilengitide is efficiently delivered into tumor	[133]

Reardon 2008	Phase II	81 GBM patients	Evaluate activity and safety in patients with GBM at first recurrence	Recurrent GBM	Single agent 500 or 2000 mg	Excellent drug safety profile	[134]
						Better antitumor activity at 2000 mg	
						PFS-6: 15%	
						OS: 9.9 months	

Nabors 2009	Phase II	112 GBM patients	Determine safety and OS	Newly diagnosed GBM	Cilengitide (500 or 2000 mg) + TMZ + RT	Well tolerated therapy	[136]
						OS: 18.9 months	
						OS at 12 months: 79.5%	

Stupp 2010	Phase I/IIa	52 GBM patients	Determine safety and efficacy of treatment	Newly diagnosed GBM	Cilengitide (500 mg) + TMZ + RT	PFS-6: 69%	[137]
						PFS-12: 33%	
						OS: 16.1 months	
						OS at 12 months: 68%	
						OS at 24 months: 35%	
						Longer PFS and OS for patients with MGMT promoter methylation	

a Cilengitide was administered i.v. twice weekly

*PK* pharmacokinetics; *DLT* dose limiting toxicity; *CR* complete response; *PR* partial response; *SD* stable disease; *OS* overall survival; *MTD* maximum-tolerated dose; *MRI*; magnetic resonance imaging; *GBM* glioblastoma; *ITH* intratumoral hemorrhage; *PFS-n* progression-free survival rate at n months; *op*. operation; *TMZ* temozolomide; *RT* radiation therapy; *MGMT O^6^*-methylguanine-DNA methyltransferase.

**Table 5:** Clinical Trials of Cilengitide Currently in Progress

Trial	Estimated no. Patients	Disease Setting	Purpose/Treatment	Start Date	Estimated Study Completion	Estimated Primary Completion	Ref.
Phase III CENTRIC	504	Newly diagnosed GBM (Methylated gene promoter status)	Evaluate safety and efficacyCilengitide + TMZ+ RT	September 2008	June 2016	September 2012	[139, 140]
Phase II CORE	264	Newly diagnosed GBM (Unmethylated gene promoter status)	Evaluate safety and efficacy Cilengitide + TMZ+ RT	December 2008	---	December 2012	[140, 141]
Phase I CILENT-0902	40	Diffuse intrinsic pontine glioma	Evaluate safety and PKCilengitide + RT	July 2010	July 2015	July 2012	[140]
Phase II ExCentric	48	Newly diagnosed GBM (Unmethylated gene promoter status)	Evaluate safety and efficacy Cilengitide + RT+ TMZ + PCB	November 2009	November 2011	January 2014	[140]
Phase II Cecil	108	Newly diagnosed GBM (Unmethylated gene promoter status)	Evaluate safety and efficacy Cilengitide or Cetuximab + RT + TMZ	September 2009	---	September 2011	[140]
Phase I	52	Progressive/recurrent GBM	Evaluate safety and dosageCilengitide + Cediranib maleate	March 2010	---	June 2010	[140]
Phase I/II CERTO	189	Advanced NSCLC	Evaluate safety and efficacy Cilengitide + Cetuximab + platinum-based chemotherapy	February 2009	November 2011	September 2011	[140, 143]
Phase I	24	Locally advanced NSCLC	Evaluate MTD Cilengitide + Radio/chemotherapy	March 2010	August 2013	August 2012	[140]
Phase I/II ADVANTAGE	195	Recurrent/metastatic SCCHN	Evaluate safety and efficacy Cilengitide + Cisplatin + 5-FU + Cetuximab	September 2008	August 2012	January 2010	[140, 143]
Phase II IRB 2004-697	106	Metastatic prostate cancer	Evaluate safety and efficacyCilengitide as single agent	April 2005	December 2011	October 2007	[140, 144, 145]
Phase II UMCC 2004.045 IRB 2004-731	32	Non-metastatic prostate cancer	Evaluate safety and efficacyCilengitide as single agent	January 2005	December 2016	February 2008	[140, 144]
Phase I CIRAB	21	Brain metastases from lung cancer	Evaluate DLT and MTDCilengitide + RT	December 2008	December 2011	December 2011	[140]

*GBM* glioblastoma; *TMZ* temozolomide; *RT* radiation therapy; *PK* pharmacokinetics; *PCB* procarbazine; *NSCLC* non-small cell lung cancer; *MTD* maximum-tolerated dose; *SCCHN* squamous cell carcinoma of the head and neck; *5-FU* 5-fluorouracil; *DLT* dose limiting toxicity.
